# Proteogenomic annotation of T6SS components identified in *Bacteroides fragilis* secretome

**DOI:** 10.3389/fmicb.2025.1495971

**Published:** 2025-02-11

**Authors:** Natalya B. Zakharzhevskaya, Olga Yu Shagaleeva, Daria A. Kashatnikova, Anton O. Goncharov, Daria V. Evsyutina, Dmitry A. Kardonsky, Elizaveta A. Vorobeva, Artemiy S. Silantiev, Viktoria D. Kazakova, Irina V. Kolesnikova, Ivan O. Butenko, Anna A. Vanyushkina, Svetlana V. Smirnova, Andrei V. Chaplin, Boris A. Efimov

**Affiliations:** ^1^Lopukhin Federal Research and Clinical Center of Physical-Chemical Medicine of Federal Medical Biological Agency, Moscow, Russia; ^2^The Laboratory of Ecological Genetics, Vavilov Institute of General Genetics, Russian Academy of Sciences, Moscow, Russia; ^3^Department of Microbiology and Virology, Pirogov Russian National Research Medical University, Moscow, Russia; ^4^Research Institute for Systems Biology and Medicine, Moscow, Russia; ^5^Vladimir Zelman Center for Neurobiology and Brain Rehabilitation, Skolkovo Institute of Science and Technology, Moscow, Russia

**Keywords:** vesicles, immune and effector proteins, T6SS, proteome, *Bacteroides fragilis*

## Abstract

**Introduction:**

*Bacteroides fragilis* (Bf)’s T6SS locus has been characterized and shown to have functional activity in competition experiments. It has been demonstrated that symbiont nontoxigenic Bf strains have a more effective “weapon” effect on pathogenic Bf, which is realized through the activity of effector-immune (E-I) protein pairs. Intensive study of the T6SS structure has led to an understanding of certain issues related to its functional activity, but the exact regulatory mechanisms of E-I protein pair activity remain unclear. Proteomic annotation of T6SS components and detailed descriptions of all immune-effector pairs are currently available. In this research, we performed detailed proteogenomic analysis and subsequent proteomic annotation of the T6SS components of the toxigenic Bf BOB25.

**Material and methods:**

Fractionated cells, cultivated media and vesicles were prepared for proteome analysis by HPLC-MS/MS. Proteogenomic annotation and comparative genomic study of the T6SS loci of the toxigenic Bf BOB25 were carried out by comparison with the reference genomes of the following Bf strains: JIM10, NCTC 9343 and 638R.

**Results:**

According to the data obtained, T6SS components were represented in all types of the analysed samples. The following components of the T6SS were identified in culture media and cells: ClpV (TssH), TssK, TssC, TssB, Hcp (TssD), and TetR. The predicted effector protein AKA51715.1 (VU15_08315) was also detected in media. The greatest amount of T6SS proteins, including the Hcp protein, was detected in the vesicle samples, which was also observed by TEM. Potential effectors, including AKA51715.1 (VU15_08315), AKA51716.1 (VU15_08320), AKA51728.1 (VU15_08385) and the immune protein AKA51727.1 (VU15_08380), were detected in vesicles.

**Discussion:**

The presence of the immune and effector proteins in the Bf secretome indicates the high activity of the T6SS without bacterial competition. It is possible that the T6SS is also used by bacteria to regulate population size by altering the activity of different repertoires of E-I pairs.

## Introduction

Bacteroides are the most represented gut anaerobes, accounting for 20–80% of the fecal microbiota (Arumugam et al., 2011). Among the commensal microbiota, Bacteroides are involved in the fermentation of carbohydrates, proteolytic degradation of proteins, and biotransformation of bile acids (Gibson and Macfarlane, 1988). Some Bacteroides species have a significant impact on the functional activity of the host immune system, protect the gastrointestinal tract from pathogen colonization, and maintain intestinal homeostasis (Wexler and Goodman, 2017; Troy and Kasper, 2010). The functional activity of the genus Bacteroides is limited to a range of bacterial secretion systems (Robitaille et al., 2023). The ability of Bacteroides to secrete outer membrane vesicles (OMV), which contain polyfunctional enzymes and metabolites, is well known (Zakharzhevskaya et al., 2017; Reeves et al., 1997; Elhenawy et al., 2014). Polysaccharide A is located on the surface of *Bacteroides fragilis* (Bf) vesicles, which contribute to immune response suppression during intestinal inflammation (Shen et al., 2012). On the other hand, Bacteroides utilize the type VI secretion system (T6SS) for bacterial competition (Bongiovanni et al., 2024). T6SS mediates cell contact-dependent intra-and inter-species bacterial antagonism (Russell et al., 2014). The T6SS pathway requires the functions of 13 core proteins, and unique subsets of these proteins appear to be evolutionarily related to T4SS components or bacteriophages (Cascales and Cambillau, 2012; Boyer et al., 2009; Gallique et al., 2017). TssL and TssM proteins, similar to those in the T4SS, are integral membrane proteins that form a transmembrane complex with TssJ, which is an outer membrane lipoprotein of the T6SS (Coulthurst, 2013; Cianfanelli et al., 2016; Basler et al., 2012). The TssC protein in combination with TssB forms a mobile filament structure that has the same structural similarity as the bacteriophage tail (Brunet et al., 2014). VgrG is a component of T6SS, similar to the structural proteins of bacteriophages, which interact with effectors through conserved adapter domains. The Hcp protein is ring-shaped and forms a “syringe.” Hcp interacts with effectors inside its pores as a chaperone. VgrG and Hcp interact with effectors and determine pathways for their export through T6SS. Effectors are proteins that exert toxic effects on attacked cells (Hachani et al., 2016; Hachani et al., 2014; Ma et al., 2014). Known T6SS effectors include cell wall-degrading enzymes (lysozymes and glycosaminidase), nucleases, pore-forming proteins (PFTs), membrane-degrading enzymes (phospholipases, amidases, and lipases), and NAD(P) + glycohydrolase (Russell et al., 2013; Russell et al., 2011; Russell et al., 2014; Russell et al., 2012). In addition to effectors that damage target cells, T6SS encodes immune proteins that have inhibitory effects on effectors, thus protecting the attacking cell from its own effectors. Immune proteins also protect cells from the effectors of other bacterial species, contributing to bacterial species dominance in the microbiota (Chatzidaki-Livanis et al., 2014). As a rule, a pair of effector and immune proteins (E-I) are encoded adjacently within the same operon (Benz and Meinhart, 2014).

The Bf T6SS gene locus was divided into three genetic architectures (GAs): GA1, GA2, and GA3. All GA loci have highly homologous segments, including the main genes encoding the Tss proteins (Coyne et al., 2016). The GA1 and GA2 loci are located on integrative conjugative elements (ICEs) and contain genes that encode known toxic effectors and related immune proteins. Recently, published data have shown that GA3 is specific only to *B. fragilis*. GA3 does not contain a conserved ICE, and is therefore not readily transferred between Bacteroidales species (Verster et al., 2017). The authors showed that the effector and immune proteins of the GA3 T6SS region are encoded by two variable regions. According to available research, Bf T6SSs are able to target most human gut Bacteroidales strains that do not contain cognate immune proteins. Functional analysis of Bf NCTC 9343 revealed that the genes BF9343_1937 and BF9343_1928 encode effectors. The protein encoded by BF9343_1928 contains the MIX domain found in other T6SS effector genes. Thus, some effector proteins can be predicted by the presence of characteristic domains. Genes encoding immune proteins are located upstream of the effector genes. BF9343_1936 (later named bti1 and Bf immunity 1) and BF9343_1927 – BF9343_1926 (similarly, bti2a and bti2b) were found to encode immune proteins. In addition, BF638R_1987 and BF638R_1978 have been identified as immune protein genes (Chatzidaki-Livanis et al., 2016).

In our study, we focused on the functional activity of T6SS outside of interbacterial competition. Taking into account already annotated proteins from the variable regions of a number of Bf strains, we carried out functional annotation of structural and functional proteins for the toxigenic Bf BOB25.

## Results

### Proteogenomic annotation of the T6SS in *Bacteroides fragilis* BOB25

**Figure 1:**
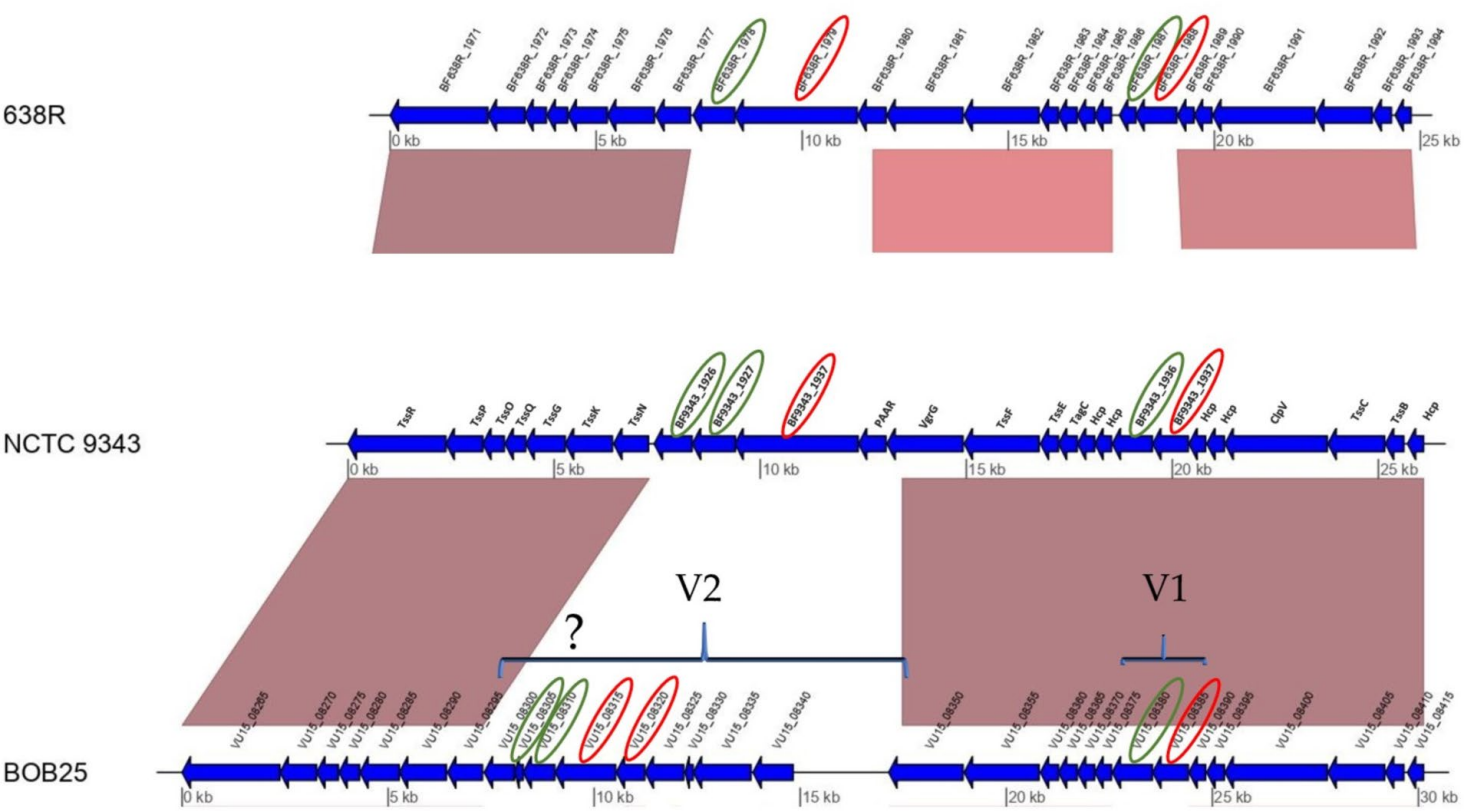
A genome comparison and proteogenomic annotation of Bf 638R, Bf NCTC9343, and Bf BOB25 revealed putative E-I pairs. Putative immune proteins and effectors are circled in green and red, respectively. The identification of immune proteins in the V2 region indicates that the activity of these proteins has yet to be proven through functional tests.

The two most studied Bf strains, NCTC 9343 and 638R, were used for genome comparisons with Bf BOB25 (Supplementary Figure S1). Proteogenomic annotation of the GA3 locus in the Bf BOB25 strain was also performed. Bf BOB25 is a toxigenic strain that has been previously sequenced (Nikitina et al., 2015). According to the data obtained, extended fragments of the GA3 locus had regions homologous to all compared Bf strains, including Bf BOB25. In particular, all structural components of the T6SS of the analyzed Bf BOB25, NCTC 9343, 638R, and JIM10 completely coincide. The GA3 locus of T6SS was constructed using the Cor-forming transmembrane proteins TssR, TssP, TssO, TssQ, TssG, TssK, TssN, TssC, and TssB. The T6SS syringe base-forming proteins included TssF, TssE, TagC, and Hcp. Two variable regions, V1 and V2, were observed for all the analyzed strains, including Bf BOB25, as shown in Figure 1. Toxigenic Bfs NCTC 9343 and BOB25 were largely genetically homologous, including the V1 region. Several genes in the V1 region were also aligned with Bf Jim10 and Bf 638R. The most extended V2 region was observed in Bf BOB25 and Bf JIM10. Bf 638R and Bf NCTC 9343 contained only to 3–4 genes in the V2 region. Proteogenomic annotation allowed us to estimate the size of the V2 region at the T6SS locus of Bf BOB25 and Bf JIM10. According to the data obtained, V2 in Bf BOB25 and Bf JIM10 contained 7–9 genes encoding proteins of unknown function. Some genes in Bf BOB25 and Bf JIM10 coincided with the V2 region. In particular, the VU15_08320 gene of Bf BOB25 almost completely overlapped with the BCV58_RS00125 gene of Bf JIM10. The VU15_08325, BCV58_RS00130, and BCV58_RS00135 genes also partially overlapped. VU15_08340 and BCV58_RS00140 had homologous fragments. No further homologous fragments were identified during the comparison of strains Bf BOB25 and Bf NCTC 9343 or Bf 638R and Bf JIM10.

Variable regions usually encode immune defense proteins and effectors. The GA3 proteogenomic study allowed us to identify genomic regions that encode proteins with unknown function. Bf BOB25 contained VU15_08380 and Vu15_08385 genes in the V1 region, which were genetically related to the BF9343_1936 and BF9343_1937 genes of Bf NCTC 9343. These genes have previously been shown to encode a functionally active pair of immune and effector proteins (Chatzidaki-Livanis et al., 2016). The V2 region of Bf NCTC 9343 contains another pair of immune and effector proteins, BF9343_1926(27) and BF9343_1928. It can be assumed that the V2 region of Bf BOB25 also contains immune and effector proteins.

**Figure 2:**
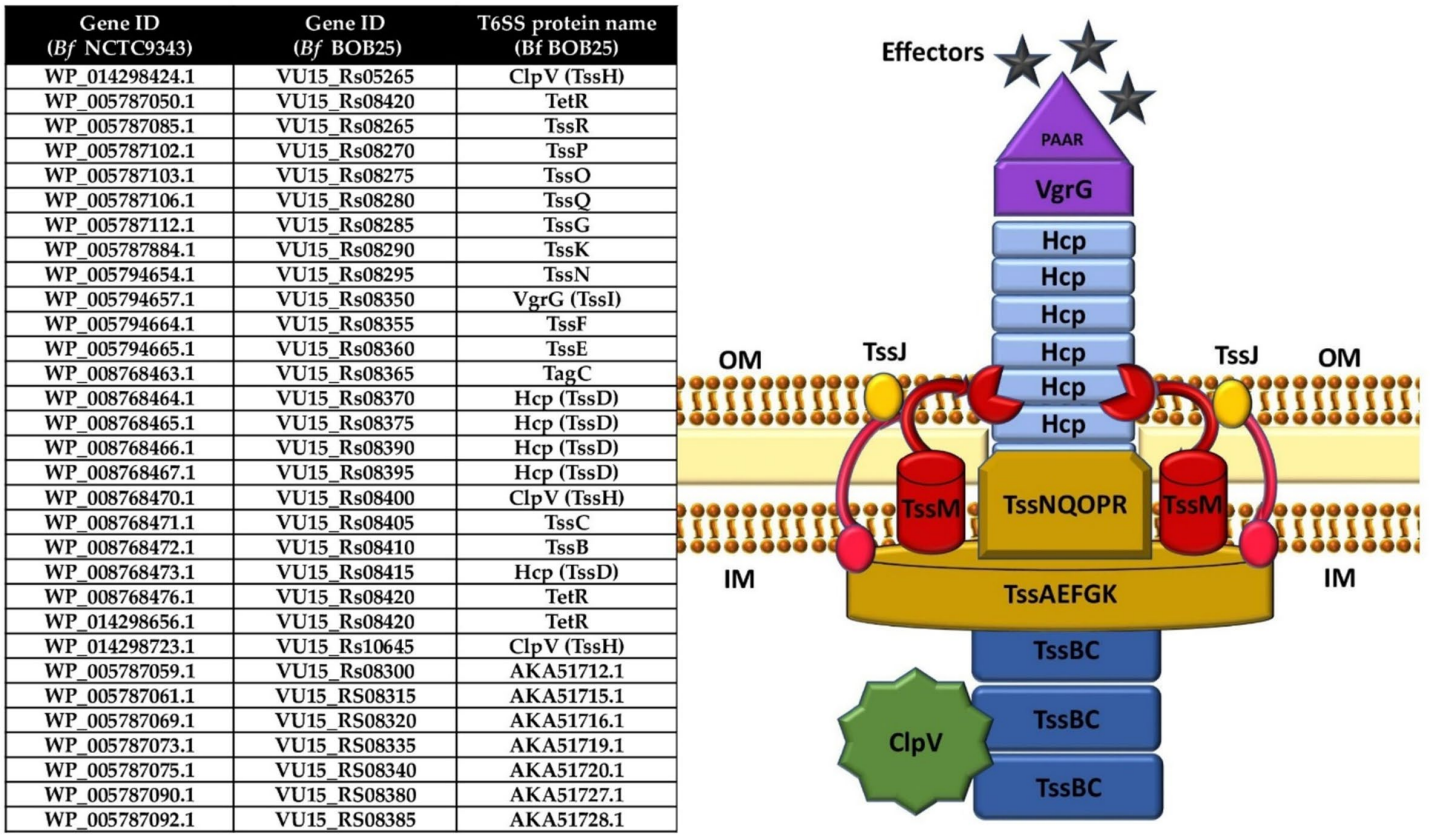
T6SS structure components annotated by proteogenomic analysis. Table contain genes ID for NCTC 9343 and BOB 25 Bf strains corresponding to the available protein’s names. The image clearly shows the T6SS structure according to the protein composition.

The remaining T6SS proteins of Bf BOB25 were fully annotated by proteogenomic analysis (Figure 2). In the next stage, functional annotation of putative effector and immune proteins encoded in variable regions (V1 and V2) was performed. We also characterized protein homologs that are encoded in variable regions. Detailed information is presented in Supplementary Table 1.

### Functional annotation of immune-effector protein pairs for the bf BOB25

Proteins from the V1 region were functionally annotated. It was previously shown that the Vu15_08380 and Vu15_08385 genes of Bf BOB25 and the BF9343_1936 and BF9343_1937 genes of Bf NCTC 9343 were highly homologous, indicating a potential overlap of protein functions (Figure 1). BF9343_1936 encodes an immune protein, whereas BF9343_1937 encodes an effector. Consequently, VU15_08380 and Vu15_08385 of Bf BOB25, which align with these fragments, are likely responsible for these functions. BF9343_1936 differs from VU15_08380 by only one amino acid substitution (K130≥I). According to several published functional tests, genes that encode effector and immune proteins are located near the structural elements of T6SS components. However, the functional annotation of E-I protein pairs is of great interest.

Most of the annotated effectors are cell membrane-degrading enzymes, and some interact with DNA to promote degradation. Annotation of the functional domains of effector and immune proteins may contribute to the functional characteristics of the protein pairs. Functional annotation of proteins encoded by the Vu15_08380 and Vu15_08385 genes was performed using the available protein function prediction databases. The DUF4595 domain was detected in the AKA51727.1 protein encoded by the Vu15_08380 gene, which is potentially an immune protein. A similar domain was detected in the protein encoded by the BF9343_1936 gene of Bf NCTC 9343. According to the obtained data, the Vu15_08380 gene encodes the lipoprotein AKA51727.1, which is highly homologous to the A0A5N0LCN4-DUF4595 domain-containing protein of *Bacteroides xylanisolvens* (H204; BIOML-A62; BIOML-A74). A homolog of the AKA51727.1 protein is also found in various Bf strains (12,905; HAP130N_ 3 B; BF_DU_COP_DK_1981). It can be assumed that this protein is a universal form of bacterial defense against Bacteroides species.

The Vu15_08385 gene encodes the AKA51728.1 protein of Bf BOB25, and it is possibly an effector with a conserved structure. This protein is often present in various Bacteroides species and functions as an effector protein. According to the predicted data, AKA51728.1 contains domains with DNA-and protein-binding abilities. By functional annotation using the GO prediction resource (ProteInfer), potential homologs of this protein were shown to be involved in the following pathways: GO:0051172, negative regulation of nitrogen compound metabolic processes; GO:0031327, negative regulation of cellular biosynthetic processes; and GO:0004659, prenyltransferase activity. In particular, several close homologs of this protein are involved in the negative regulation of protein translation activity.

**Figure 3:**
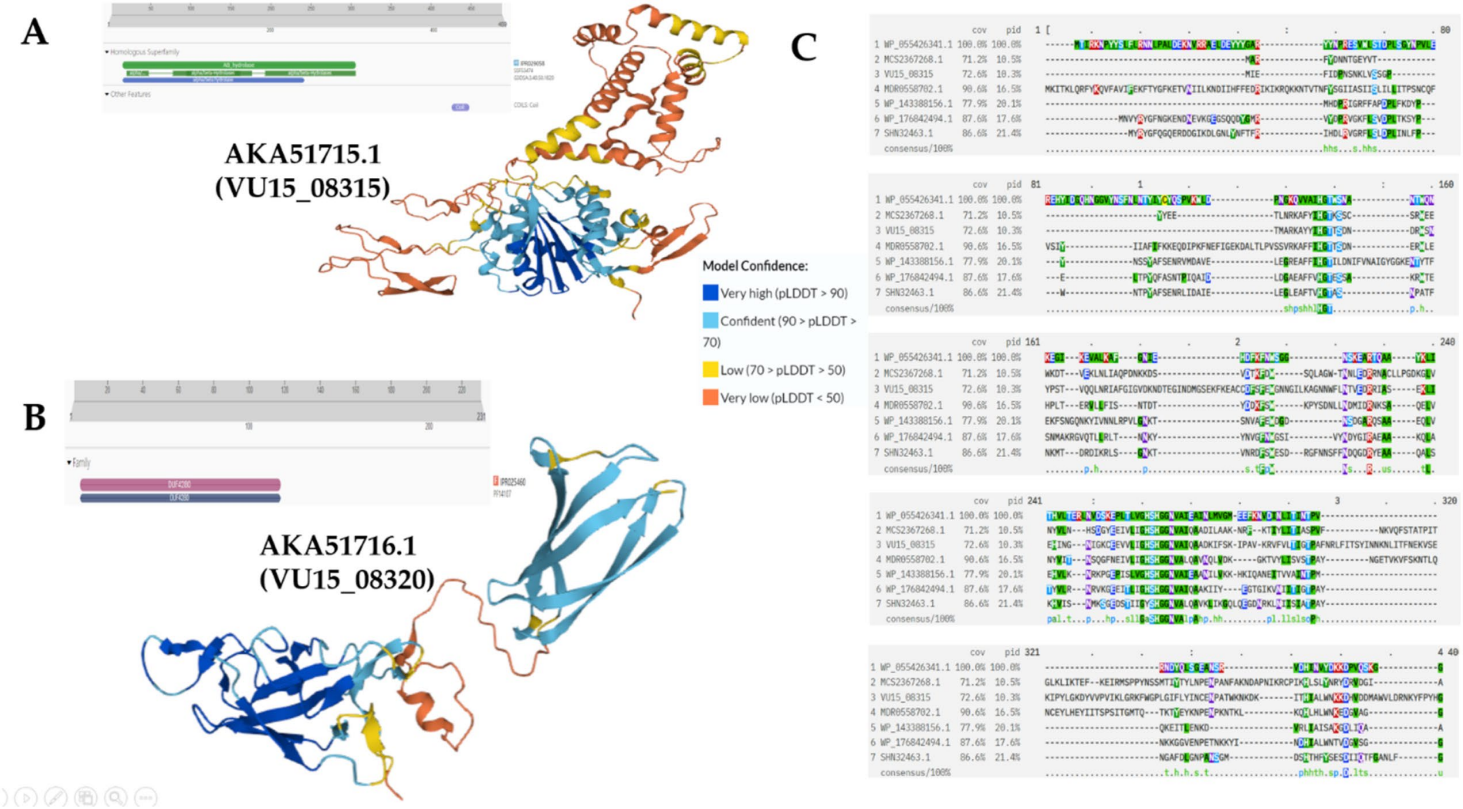
Functional annotation of the E-I protein pairs in the V2 region. **(A)** Structure of the AKA51715.1 protein revealed the presence of motifs composed of serine, aspartate, glutamine, and lysine, which are active amino acids. Functional annotation of the AKA51715.1 protein revealed the presence of alpha/beta hydrolase domains. **(B)** Structure of the AKA51716.1 protein with the predicted DUF4280 domain. **(C)** Genome alignment of the AKA51715.1 protein with its distant homologs was performed via Psi-BLAST.

Next, the proteins encoded in the V2 region (VU15_08300; VU15_08315; VU15_08320; VU15_08335; and VU15_08340) were functionally annotated (Figure 3; Supplementary Figure S2). VU15_08315 encodes the AKA51715.1 protein located in the V2 region. Functional annotation of the AKA51715.1 protein revealed the presence of DUF2974 and RHS repeat-associated core domain motifs, which can be found in various T6SS effector proteins in different bacterial species (Figure 3A). The AKA51715.1 protein also contains a hydrolase domain, which may indicate the presence of enzymatic activity in this protein (Figure 3A). Potential homologs of this protein are presented in Supplementary Table 1. When searching for distant homologs using the Psi-BLAST service, the following proteins were selected: MCS2367268.1 DUF2974 domain-containing protein (*Bacteroides caccae*), MDR0558702.1 MAG: DUF2974 domain-containing protein (Prevotellaceae), and WP_176842494. 1 RHS repeat-associated core domain-containing protein (*Chitinophaga filiformis*); WP_055426341.1 RHS repeat-associated core domain-containing protein (Apibacter mensalis); WP_143388156.1 Mbeg1-like protein (Flavobacterium gawalongense); SHN32463.1 Putative serine esterase (*Cyclobacterium lianum*). Conserved amino acids and whole motifs composed of active amino acids, serine, aspartate, glutamine, and lysine, were observed during the alignment (Figure 3C). The presence of a large amount of histidine may indicate a zinc-or iron-binding ability. Additional annotation using ProteInfer suggested several potential pathways in which this protein may be involved: GO:0044238, primary metabolic process; GO:0044424, intracellular part; GO:0019752, carboxylic acid metabolic process; and GO:0016829, lyase activity. Thus, AKA51715.1, protein is highly likely to be an effector protein. Its presence in the Bf secretome as well as the detection of the effector protein AKA51728.1, encoded by the VU15_08385 gene, may indicate the presence of several simultaneously activated E-I protein pair repertoires within the same population.

VU15_08320 encodes AKA51716.1, which is also located in the V2 region. Functional annotation of the AKA51716.1 protein demonstrated the presence of the DUF4280 domain (Figure 3B). According to Rigard’s research, the effector protein may contain a DUF4280 domain (renamed in the paper PAAR-like domain). M. Rigard showed that the DUF4280 domain-containing protein of Francesella sp. is a metal-binding protein that, based on its PAAR-like domain, might cap the VgrG spike of the T6SS and act as a membrane-puncturing protein (Rigard et al., 2016). Prediction using ProteInfer did not add additional data about AKA51716.1 protein functional activity, indicating that only the most common pathways in which this protein may be involved were GO:0005737: cytoplasm; GO:0046872: metal ion binding; and GO:0009987: cellular process. Interestingly, J. Wang also showed that DUF4280 domain-containing proteins are potential effectors for other bacterial species (Zhang et al., 2021).

The VU15_08335 gene, which encodes AKA51719.1, was functionally annotated. According to the data obtained, the AKA51719.1 protein is completely homologous to lipoprotein A0A2M9V3N9 of Bf 12,905 and protein R7JKL0, which is closely related to the *Alistipes putredinis* CAG:67 of MGS:67. This protein contains several domains (1–230 and 231–466), one of which is the transmembrane domain (Supplementary Figure S2). The AKA51719.1 protein is most likely a membrane-associated protein. According to the results of the GO enrichment analysis (ProteInfer), close homologs of the AKA51719.1 protein are involved in the following pathways: GO:0003824, catalytic activity; GO:0005975, carbohydrate metabolic process; and GO:0009987, cellular process. There is no obvious enzymatic function for this protein, thus the probability that this protein is an effector is very low. It is not possible to assume that this protein is immune based on available information.

Functional annotation of AKA51720.1, encoded by the VU15_08340 gene, revealed that this protein is a lipoprotein and is highly conserved throughout the Bacteroides genus. A well-defined signal peptide was detected in the protein structure (Supplementary Figure S2). This protein is likely membrane-bound, and AKA51719.1 protein does not appear to be an effector protein. According to GO prediction (ProteInfer), close homologs of this protein are involved in the following pathways: GO:0044464: cell part; GO:0003674: molecular function.

The other proteins within the V2 variable region, encoded by the VU15_08300, VU15_08305, and VU15_08310 genes, were also functionally annotated. AKA51712.1 protein encoded by VU15_08300 is a CHAT domain-containing protein that appears to be a transmembrane protein (Supplementary Figure S2). The AKA51713.1 proteins encoded by VU15_08305 was very short and could not be annotated functionally. The AKA51714.1 proteins encoded by VU15_08310 protein is also a CHAT domain-containing protein. Since these proteins are located in close proximity to effectors, there is a high probability that they are immune proteins. However, given that most pathogenic bacteria lose interspecies competition, it is likely that only effectors are present in the variable region.

Next, the abundance of T6SS proteins was assessed via proteomic analysis of bacterial cells, media, and vesicles. Proteome analysis mainly focused on searching for proteins in the V1 and V2 regions, which were annotated by proteogenomic and functional analyses.

### Proteomic profiling of T6SS structural and effector–immune pair proteins

**Figure 4:**
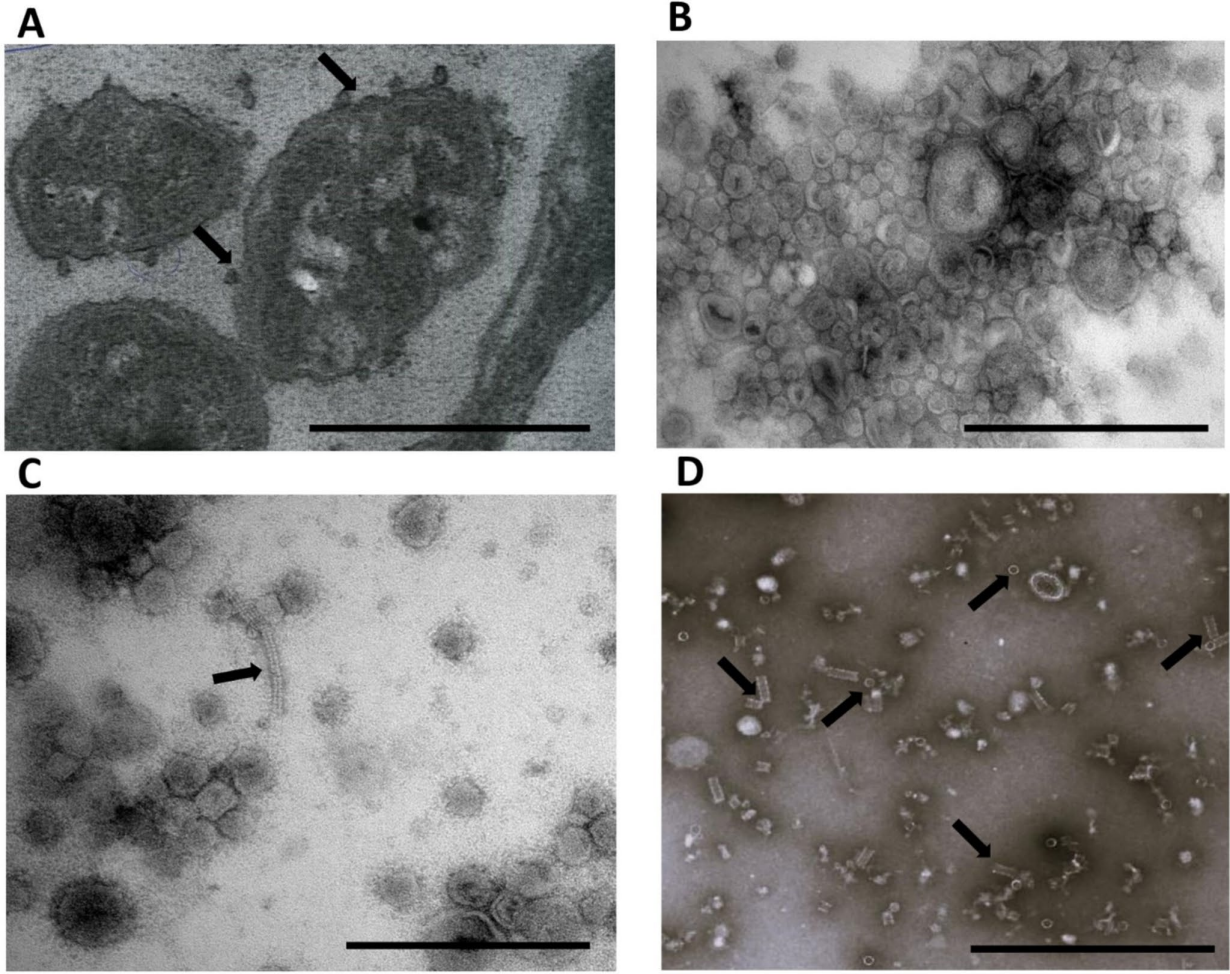
*Bacteroides fragilis* BOB25 produces outer membrane vesicles and demonstrate the T6SS activity. **(A)** TEM of thin sections of Bf BOB25 cells during vesicle production. Arrows indicate vesicles secreted by Bf. Scale bars represent 2 μm. **(B)** Isolated Bf BOB25 vesicles. The scale bars represent 500 nm. **(C)** The vesicle sample was diluted 10-fold and contained the Hcp protein constructed in a syringe, as indicated by the arrow. Scale bars represent 500 nm. **(D)** A vesicle sample diluted 1,000 times containing individual rings of the Hcp protein and Hcp protein constructed in a syringe. The rings and syringe structure are indicated by arrows. Scale bars represent 500 nm. A-D at magnification: x10,000.

To assess the functional activity of the T6SS in Bf BOB25, the following samples were prepared: bacterial cells, culture media and bacterial outer membrane vesicles (OMVs). Culture media were purified from the cells, cell fragments, and OMVs by filtration and ultracentrifugation. All the samples were examined using electron microscopy. The prepared sections contained bacterial cells that actively produced OMVs (Figures 4A,B). OMV and media samples were evaluated separately. Fragments of the T6SS-Hcp protein, which were observed in small amounts in media samples, were clearly observed. However, the OMV samples contained a significant amount of Hcp protein, both as independent rings and assembled syringe structures. The diluted OMV samples were more convenient for Hcp protein analysis (Figures 4C,D). Additionally, supernatant obtained after ultracentrifugation were prepared for proteome analysis. According to the data obtained, sample did not contain T6SS components and cytoplasmic proteins. The NTA method was used to assess OMV size and concentration. According to the NTA data, the average size of the Bf BOB25 OMVs ranged from 60 to 150 nm. However, it was not possible to assess the size of the Hcp protein ring. However, according to literature, the Hcp protein has an average size of approximately 15–20 nm, which is clearly visible in the obtained images. Similar TEM images have been presented in various publications devoted to the T6SS analysis (Zoued et al., 2014; Del Tordello et al., 2016; Nazarov et al., 2018). It was not possible to resolve the other components of the T6SS using the proposed method. OMV concentration measured using NTA was approximately 9 × 10^13^ particles/ml.

The isolated and purified OMVs, as well as the bacterial cells and cultivated media, were analyzed by HPLC-MS/MS. Bacterial cells in the stationary growth phase were removed when an optical density of D = 0.9 was reached. The media were completely removed from the bacterial cells by filtration and ultracentrifugation. The media sterility was checked separately by the absence of culture growth on solid media. OMVs were isolated according to a preestablished protocol.

**Figure 5:**
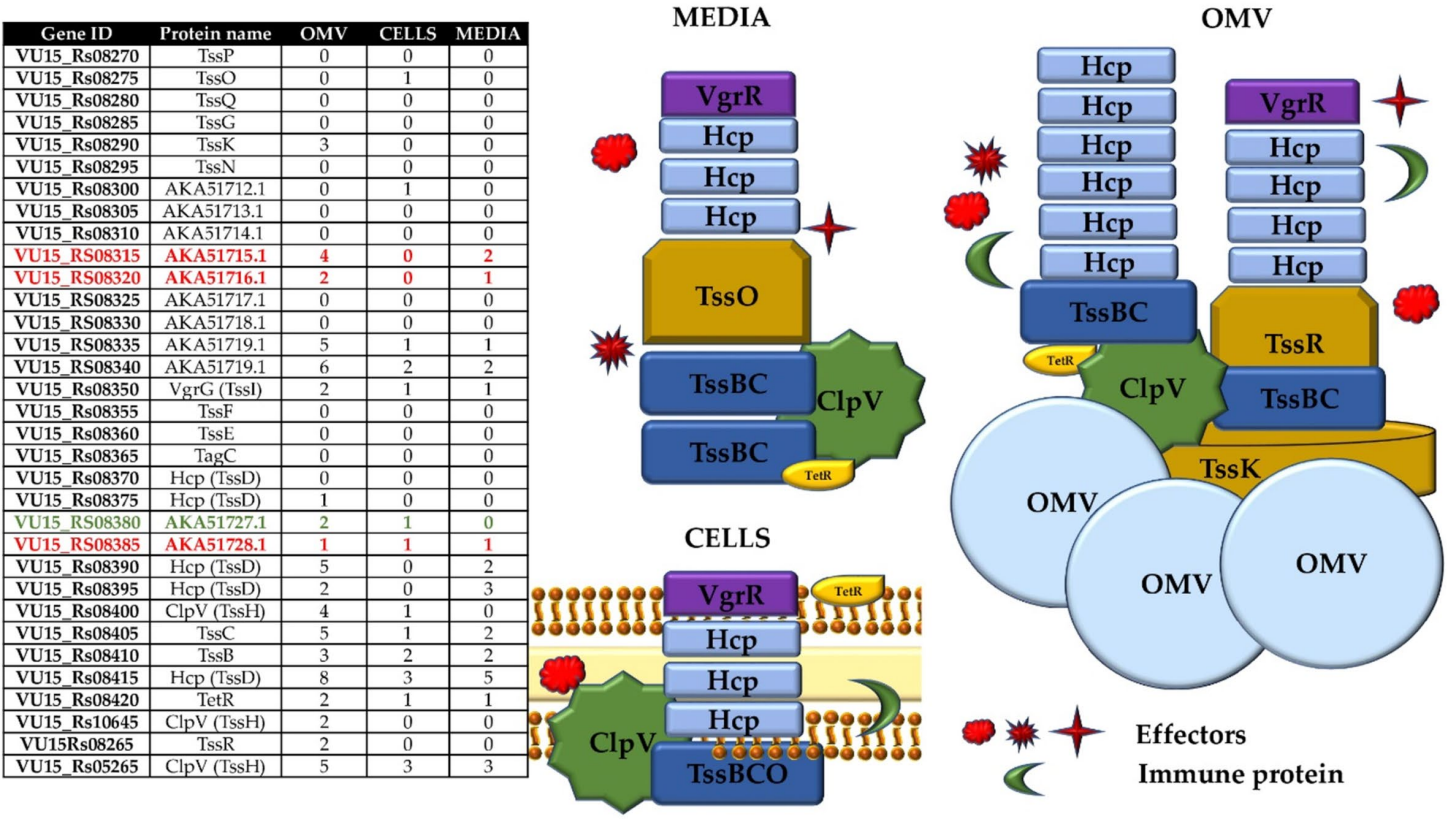
Complete list of the identified T6SS proteins of *Bacteroides fragilis* BOB25 in all types of samples. The figures fully reflect the types of proteins that were identified in the samples. Potential effectors and immune proteins are colored in red and green in table, respectively.

Analysis of the bacterial cell proteome was performed as the combined proteome of the membrane and cytoplasm cell fractions. As a result, 51 HPLC–MS/MS runs were carried out during comprehensive proteome analysis of the abovementioned samples. In total, 17,644 unique peptides were identified against the database constructed from Bf BOB25 proteins (ProtDB). Additionally, 1,465 proteins were identified by 2 or more peptides across all experiments (cells, media and OMVs). The complete list of the identified peptides and proteins, the score thresholds and FDRs are provided in Supplementary Table 2. These tables contain information about the peptide sequence, emPAI and number of identified unique peptides per protein. To identify novel proteins inside the GA3 region, we performed a proteogenomic approach using a database generated from a six-frame translation of the genome sequence for our search. Alternate names starting with AKA are previously unannotated proteins belonging to the T6SS. Proteomic profiling of the media, vesicles and cell samples revealed a significant number of proteins. On average, approximately 160 proteins were annotated in the vesicle samples, approximately 650 proteins were found in the bacterial cell lysate samples, and 130 proteins were found in the bacterial media. Particular attention was given to the identification of the proteins of the T6SS. This was not expected, but components of the T6SS were found in all types of analyzed samples (Figure 5).

Based on the TEM data, the main components of the syringe, the Hcp protein, were identified. However, the spectrum of identified T6SS proteins was more significant. The largest number of T6SS structural proteins was identified in the OMV samples. As shown in Figure 5, almost all components of the T6SS, including membrane proteins that form the core of the T6SS, were identified in the OMV samples. A small portion of T6SS proteins were identified in cells, media and OMVs. The following proteins of the T6SS tail complex were found: TssK, TssF, TssG, TssE, TssB and TssC. The enzyme ClpV was also detected in OMV samples. ClpV cleaves the interconnected proteins TssB and TssC when the syringe is released into the external environment. The TssB and TssC proteins were detected in all sample types, and TssK was detected in OMV and media samples. The TssP, TssQ, TssF, TssG, and TssE proteins were not detected. Most likely, when the T6SS shell is destroyed, free proteins enter both the cell (or the vesicle when the T6SS apparatus is located in the vesicle membrane) and the external environment. Hcp and VgrG proteins were found in all sample types, while PAAR, the needle tip protein, was found only in OMV samples. This may be because the PAAR protein may degrade. TssR proteins were found exclusively in vesicle samples. Additionally, the unknown proteins TssP, TssO, TssN and TagC were not detected in the analyzed samples. The antibiotic resistance protein TetR was detected in all the samples. Proteomic profiling revealed that the main components of the T6SS were detected in vesicles and media samples. It can be concluded that the T6SS may function without any bacterial competition. It is also important to assume that T6SS structural proteins may have an expanded range of properties, which determines their presence in media and vesicle samples.

After evaluating the structural components of the T6SS in the cell, media and vesicle proteomes, we searched for immune and effector proteins in the analyzed samples. Based on the results of proteogenomic annotation and literature data, the most likely regions encoding immune and effector proteins are the genomic regions flanking the Tss D genes encoding Hcp proteins (Chatzidaki-Livanis et al., 2016). The BF9343_1936 and BF9343_1937 genes as well as the BF9343_1926 and BF9343_1927 genes were previously annotated for Bf NCTC 9343 as an E-I protein pair. According to the genome alignment data obtained for the Bf strains NCTC 9343 and Bf BOB25, the BF9343_1936 and BF9343_1937 genes are highly homologous to the Vu15_08380 and Vu15_08385 genes. We suggest that Vu15_08380 and Vu15_08385 also encode immune and effector proteins. The BF9343_1926 and BF9343_1927 genes were previously annotated as immune and effector proteins, but they did not match genes in the V2 region of Bf BOB25. However, it can be assumed that the V2 region (VU15_08295-VU15_08340) may also contain an E-I protein pair located upstream of the TssN gene. Thus, a proteomic study revealed proteins from the V1-V2 region in all types of samples. AKA51715.1 (VU15_RS8315), AKA51719.1 (VU15_RS08335) and AKA51719.1 (VU15_RS08340) were found in media samples. AKA51719.1 (VU15_RS08340), AKA51727.1 (VU15_RS08380) and AKA51728.1 (VU15_RS08385) were detected in bacterial cell lysate samples. A significant number of proteins originating from the analyzed V1 and V2 regions were identified in OMV samples: VU15_08315; VU15_08320; VU15_08335; VU15_08340; VU15_08380; and VU15_08385. Notably, the proteins VU15_08380 and VU15_08385 were detected in the cells but not in the media. It is likely that these proteins may be included in OMVs and transported to other bacterial cells. The VU15_08315 and VU15_08320 genes encode predicted effectors, and both were detected in OMV samples. Interestingly, all putative effectors were found in the OMV samples, which means that the T6SS is functionally active in a homogeneous bacterial population without any competition, which normally occurs between different bacterial species. However, we found only AKA51727.1, a predicted immune protein, in OMV and cell samples and did not find any predicted immune proteins in the V2 region. It is possible that Bf toxigenic strains obtain only genes encoding effectors in variable regions via horizontal gene transfer. However, it cannot be ruled out that the putative immune proteins are not expressed outside of interbacterial competition.

## Discussion

The gut microbiota actively utilizes the T6SS for interspecific competition (Motta et al., 2024). The T6SS has already been annotated in detail for *Bacteroides fragilis* (Chatzidaki-Livanis et al., 2016). The main mystery in T6SS function is the regulation of E-I protein pair activity. Predicting all E-I pairs for gut microbiota species is an important task that helps to better understand bacterial population shifts and may contribute to the development of new effective antibiotics. Currently, there are several ready-made solutions that can be used for predicting E-I protein pairs (Yang et al., 2018). Available data indicates that E-I protein pairs typically encode together at the GA locus and have recognizable domains (Coyne et al., 2016). Effector proteins are mainly enzymes that promote the destruction of cell membranes or influence the activity of the cell cycle by binding to DNA and RNA (Le et al., 2021). There is a lack of information regarding the functional activity of immune proteins. Immune proteins that are encoded in pairs with an effector are able to actively protect bacterial cells from their own effectors and a similar effector of the opposing cell (Hagan et al., 2023). The exact mechanism of immune protein action is not well described.

It is well known that Bfs exhibits a dual nature. Pathogenic Bfs strains have the potential to cause inflammation and cancer in the intestine (Cheng et al., 2020). Nonpathogenic strains are able to decrease inflammatory reactions and, on the other hand, guarantee intestinal recovery (Chan, 2019). In our research, we focused on the proteogenomic annotation of T6SS components of fractionated Bfs cells and secretomes.

As a result, we observed mainly membrane T6SS structures in fractionated cell samples. The cultivated media contained membrane and “syringe” T6SS proteins, and the greatest number of T6SS structural proteins were observed in the vesicle samples. The vesicle preparation protocol is also ideal for membrane-associated protein and T6SS lipoprotein isolation. Therefore, it is probably not possible to eliminate T6SS proteins when isolating vesicles, even when using gradient ultracentrifugation. TEM assay confirmed that Hcp protein were also presented in samples containing vesicles. The Hcp protein forms the basic structure of the T6SS syringe (Silverman et al., 2012). We observed Hcp in vesicle samples in assembled syringe structures and as individual “rings.” Given the fact that the T6SS syringe is morphologically similar to the bacteriophage’s components, we conducted additional proteome annotation. Bfs secretome was annotated using the self-made viral genome database made it possible to exclude the possibility of phage components contaminated vesicle samples. Type 6 secretion components were identified in all fractionated cell lysate and vesicle-containing samples. Activity of the T6SS without bacterial competition raises a number of questions. For example, what factors can provoke the activity of the T6SS in a bacterial population without competition? The most obvious seems to be the use of the T6SS for regulation of the population size. Although similar activity has been shown for other bacterial species, but it should be confirmed for Bacteroides (Silverman et al., 2012).

Proteins encoded in variable regions (V1 and V2) were the subject of our special interest. In particular, we were interested in E-I protein pair proteins detected in the secretome. We detected the AKA51727.1 and AKA51728.1 proteins encoded by the Vu15_08380 and Vu15_08385 genes, which are similar to the E-I protein pair of Bf NCTC9343 (BF9343_1936 and BF9343_1937) in fractionated cells and vesicle containing samples. The lipoprotein AKA51727.1, which was annotated as an immune protein, is conserved. This E-I protein pair (encoding the Vu15_08380 and Vu15_08385 genes) may be one of the common mechanisms of T6SS action in different Bf strains. Proteins from the V2 region were also detected in OMV samples. AKA51715.1 and AKA51716.1 proteins encoding by VU15_08315 and VU15_08320 genes were predicted to be effectors. Functional annotation of the AKA51715.1 protein revealed the presence of a DUF2974 domain, which was previously found in various T6SS effector proteins in different bacterial species (Andreia et al., 2019). The AKA51716.1 protein contained the DUF4280 domain, which is similar to the protein of Francesella sp. and might be the cap of the T6SS VgrG spike protein which acts as a membrane-puncturing protein (Lays et al., 2016). Proteins encoded by the Vu15_08305 and Vu15_083810 genes, which are located upstream of the Vu15_08315 and Vu15_083820 genes, are most likely immune proteins. In particular, the AKA51713.1 and AKA51714.1 proteins encoded by these genes contained CHAT domains, which partly unites them into common functions. However, the functional activity of each predicted effector and immune protein needs to be confirmed by competition tests. However, the annotation of these proteins in the Bf BOB25 V2 region and their secretion may indicate that the bacteria actively utilize the T6SS without any bacterial competition. Therefore, the activity of the T6SS in bacteria may be related to various adaptive factors. It can be hypothesized that bacteria may engage separate repertoires of E-I protein pairs during their functioning for the population growth suppression or as a special reaction on the changing external condition. Given the fact that immune and effectors proteins were detected in greater amounts in the OMV samples, it can be hypothesized that OMVs are part of the bacterial defense mechanism. When attacking an opponent, the bacteria engage an effector protein that is actively blocked by the immune protein of the victim bacteria. Excess immune proteins actively enter vesicles or bind to the vesicular membrane during secretion and are transported to other members of the bacterial population for passive protection.

**Figure 6:**
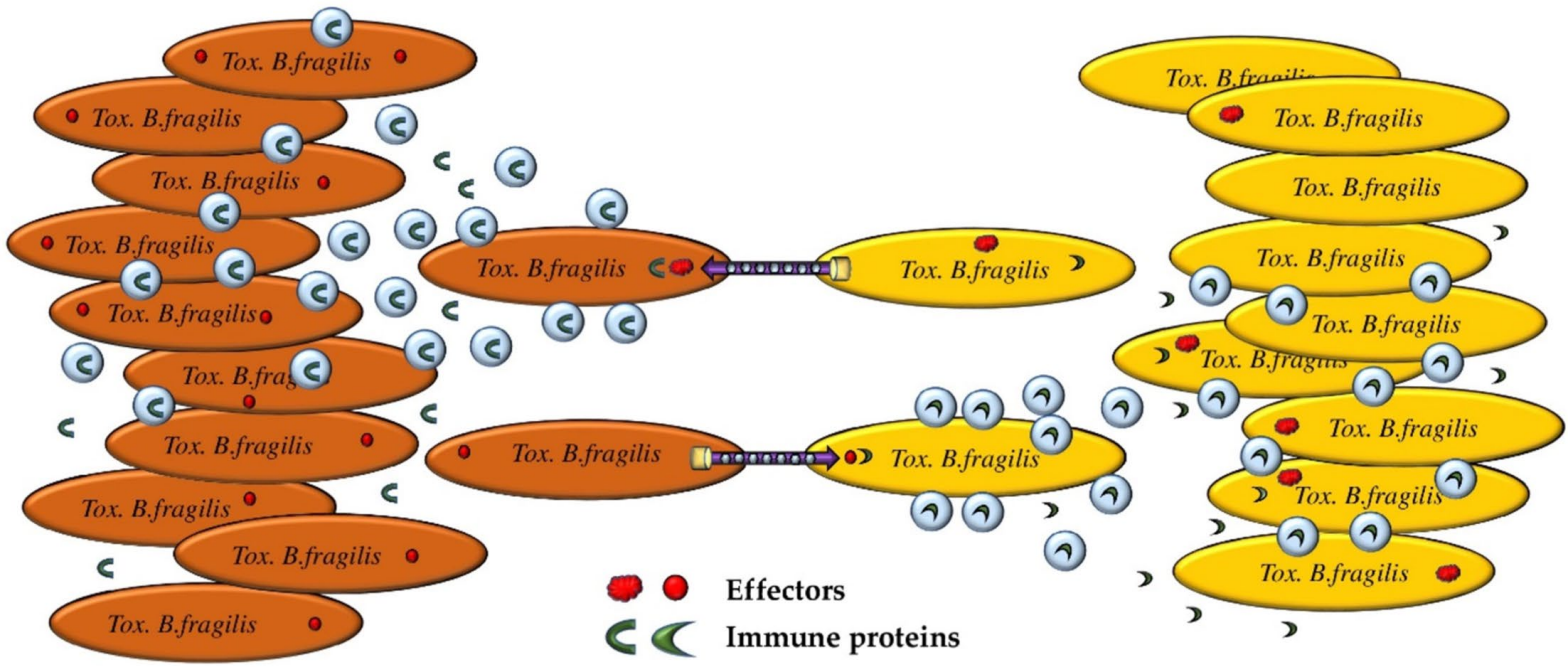
Proposed mechanism of “quorum sensing” realized by OMVs containing E-I proteins. Bacteria may express different repertoires of E-I protein pairs within the same population. With a significant increase in the bacterial population, the bacterial subpopulation is forced to actively use the T6SS to maintain the optimal population size. Depending on the variant of the active E-I protein pair, one of the bacterial subpopulations has an advantage. OMVs containing immune proteins can be used for passively or actively alerting the attacked subpopulation and preventing massive bacterial death.

The idea that a genetically homogeneous population is nevertheless heterogeneous is not new (Davis and Isberg, 2016). Bacteria have a significant number of mechanisms for adaptation. Responses to limited resources, temperature and pH changes and other vital factors determine the aggregate state of a population, which is usually assessed by determining gene expression levels, the total proteome, and other parameters (Schröter and Dersch, 2019). However, a much more important issue is the mechanisms of the formation of individual states and the adaptation of a single bacterial cell to current environmental conditions. Most likely, as a product of bacterial life activity, vesicles can be one of the adaptation mechanisms for cell–cell signaling (Figure 6).

## Conclusion

Proteome and TEM analysis with subsequent proteogenomic annotation revealed a functionally active T6SS in Bf Bob25. T6SS structural proteins, as well as immune and effector proteins, were detected in media, cell and vesicle samples. The greatest amount of T6SS proteins, including E-I protein pairs, was detected in the OMV samples. It can be assumed that the conditions for OMV and T6SS protein isolation are similar. However, it cannot be ruled out that OMVs are one of the additional mechanisms by which cell–cell signaling occurs to maintain the bacterial population size.

## Materials and methods

### Genome comparison

To identify differences between the Bf genomes (BOB25, NCTC9343, 638R and JIM10), we mapped the reads in a reciprocal way with Bowtie2. SNPs were evaluated using SAMtools mpileup and varscan with a *p* value threshold of 10^−5^, minimum four-read coverage and a frequency threshold of 0.9. Then, a custom R script was used to calculate SNP density in a sliding window of 10,000 nucleotides. The modal value was used to estimate the SNP rate. To identify unique genes, the read count per gene was calculated using bedtools, and reads with zero reads mapped to them were assumed to be unique.

### Bacterial strain and growth conditions

Enteropathogenic *B. fragilis* BOB25 (a clinical isolate kindly provided by the Lopukhin Federal Research and Clinical Center of Physical–Chemical Medicine of Federal Medical Biological Agency, Moscow, Russian Federation) was grown on blood agar plates containing either 5% defibrinated horse blood or brain heart infusion broth supplemented with hemin (5 g/mL) under anaerobic conditions established by placing Anaerogen™ bags in anaerobic 3.5 litre flasks (Oxoid; Thermo Fisher Scientific, Inc.) or in anaerobic flasks (Schuett-Biotec, Germany) at 37°C until stationary phase. For liquid culture, a preculture of BOB25 strain was grown anaerobically in Columbia Broth Base (Hi Media, India) at 37°C until stationary phase.

### Vesicle isolation and purification

Two hundred fifty milliliters of 24-h culture of Bf BOB25 were centrifuged at 4,500 g at 4°C. To remove residual cells, the supernatant was filtered through a 0.45 μm pore membrane. The filtrate was subjected to ultracentrifugation at 100,000 g_n_ for 2 h (Optima L-90 K ultracentrifuge; Beckman Coulter). The supernatant was discarded, and the pellet was washed with sterile PBS and filtered through a sterile 0.2 μm-pore polyvinylidene difluoride (PVDF) membrane (Miltenyi GV; Millipore). The ultracentrifugation step was repeated twice. The vesicle pellet was resuspended in distilled water or 150 mM NaCl (pH 6.5). The protein concentration was quantified using a 2D-quant kit (GE Healthcare Life Sciences).

### Electron microscopy

Ultrathin sections of Bf BOB25 were prepared as previously described (Farquhar, 1956). The isolated Bf BOB25 vesicles were diluted to concentrations ranging from 2 to 5 × 10^11 particles/mL and prepared for TEM analysis as previously described (Zoued et al., 2014). Additionally, the vesicle samples were diluted 100 and 1,000 times for visual assessment of the T6SS components. Images were obtained using a JEM-1400 (Jeol, Tokyo, Japan) transmission electron microscope equipped with a Rio-9 camera (Gatan Inc., Pleasanton, CA, United States) at 120 kV.

### Nanoparticle tracking analysis

Hydrodynamic particle size distribution and concentration were measured with nanoparticle tracking analysis by Nanosight LM10 HS-BF instrument (Nanosight Ltd., Salisbury, United Kingdom) with high sensitivity camera of EMCCD type, and NTA 2.3 build 33 software. Detailed protocol of NTA assay is presented in Shagaleeva et al. (2023).

### SDS–PAGE and in-gel trypsin digestion of protein samples

Approximately 200 mL of cultivated media and supernatant obtained after ultracentrifugation was lyophilized. The isolated vesicles, cells and lyophilized media samples were mixed with a Laemmli sample buffer (1:1) containing CHAPS and separated by SDS–PAGE. Forty micrograms of each media and vesicle sample and 80–90 μg of cell lysate were boiled for 10 min prior to electrophoresis. The gels were stained with Coomassie brilliant blue. The gel was cut into small (1 × 1 mm) pieces and transferred into sample tubes. The protein disulfide bonds were reduced with 10 mM DTT (in 100 mM ammonium bicarbonate buffer) at 50○C for 30 min and subsequently alkylated with 55 mM iodoacetamide (in 100 mM ammonium bicarbonate buffer) at room temperature for 20 min in the dark. After alkylation, the gel samples were stained with 50% ACN (in 50 mM ammonium bicarbonate buffer) and dehydrated by the addition of 100% ACN. After removal of 100% ACN, the samples were subjected to in-gel trypsin digestion. The digestion buffer contained 13 ng/μl trypsin (in 50 mM ammonium bicarbonate buffer). Trypsin digestion proceeded overnight at 37°C. The resulting tryptic peptides were extracted from the gel by adding two volumes of 0.5% trifluoroacetic acid (TFA) to the samples (incubated for 1 h), followed by two volumes of 50% ACN (incubated for 1 h). Finally, the extracted peptides were dried under vacuum and redissolved in 3% ACN with 0.1% FA solution prior to LC–MS/MS analysis.

### LC–MS/MS analysis of cell, media and vesicle proteomes

LC–MS/MS analysis of tryptic peptides was carried out using an Ultimate-3000 HPLC system (Thermo Scientific) coupled to a maXis qTOF after an HDC cell upgrade (Bruker) with a nanoelectrospray source. Chromatographic separation of peptides was performed on a C-18 reversed-phase column (Zorbax 300SB-C18, 150 mm × 75 μm, 3.5 μm particle diameter, Agilent). The gradient parameters were as follows: 5–35% acetonitrile in aqueous 0.1% (v/v) formic acid, duration 120 min, and column flow rate of 0.3 μL/min. Positive MS and MS/MS spectra were acquired using AutoMS/MS mode (capillary voltage 1700, curtain gas flow 4 L/min, curtain gas temperature 170\u00B0C, spectra rate 10 Hz, 4 precursors, m/z range 50–2,200, active exclusion after 2 spectra, release after 0.5 min).

### Proteogenomic database generation

The reference protein database was downloaded in FASTA format (RefSeq: NZ_CP011073.1, 4,127 amino acid sequence). The genome was downloaded from NCBI in FASTA format (NZ_CP011073.1) and was translated into 6 frames. Stop-to-stop ORFs were exported using Artemis software version 16.0.0 with the option “Mark Open Reading Frames.” The minimal ORF length was set at 40 amino acids.

### Virome data base generation

To construct a virome database for subsequent proteomic analysis, existing bioinformatics approaches for annotation of known phages and the Refseq viral genome database were used (Wood and Salzberg, 2014; O'Leary et al., 2016). Orthologous groups of phage genes were also taken into account (Grazziotin et al., 2017).

### Protein and peptide identification

Raw data files in the WIFF and D files were converted to the Mascot generic format (MGF file format) using AB SCIEX MS Data Converter version 1.3 and Compass Data Analysis 4.2 (Build 383.1), respectively. Protein identification was carried out using Mascot Search Engine version 2.5.1. The Mascot searches were performed with the following parameters: tryptic-specific peptides, maximum of one missed cleavage, a peptide charge state limited to 1+, 2+ and 3+ a peptide mass tolerance of 10 ppm, a fragment mass tolerance of 0.5 Da, variable modifications caused by oxidation (M) and propionamide (C). The decoy search strategy for calculating the FDR was used. The score threshold was calculated using Mascot. Individual ion scores higher than the score threshold indicate identity or extensive homology, with *p* < 0.05 and an FDR < 5%. A peptide was identified if its rank was 1 and the score was higher than the score threshold. A protein was identified if it had 2 or more identified unique peptides.

### Proteogenomic analysis

MS/MS data were searched against a six-frame translated genome sequence (GenDB) to identify novel protein-coding regions. After excluding peptides identified in the protein database (ProtDB), the genome search-specific peptides (GSSPs) were further analyzed to refine the current genome annotations. GSSPs were categorized into three groups: (1) peptides mapped to an ORF that does not contain a gene, (2) peptides mapped to an ORF that contains a gene, and (3) peptides mapped to an ORF that contains a pseudogene. ORFs that contained at least 2 GSSPs and did not include genes were marked as CDS-containing regions. The start codon of the CDS, which contains at least one GSSP in the same ORF, was reannotated. CDS reannotation was carried out using the Prokka tool with default parameters. Sequencing errors were searched in regions that contained GSSP and pseudogenes on the same strand. ORFs that are in the pseudogene environment (±1,000 bp) and have the same strand were aligned to the NCBInr database using the BLASTP algorithm. ORF sequences aligned to the same protein were selected for analysis of sequencing errors and frame shifts.

## Data Availability

The datasets presented in this study can be found in online repositories. The names of the repository/repositories and accession number(s) can be found in the article/Supplementary material. The mass spectrometry proteomics data have been deposited to the ProteomeXchange Consortium via the PRIDE partner repository with the dataset identifier PXD059123.
