# Supplementary material for: Proteogenomic annotation of T6SS components identified in Bacteroides fragilis secretome
Source: Front Microbiol. 2025 Feb 11;16:1495971. doi: 10.3389/fmicb.2025.1495971 (PMC11854122; doi:10.3389/fmicb.2025.1495971)
Supplement: Supplementary file 1 [file Data_Sheet_1.docx]

Supplementary information


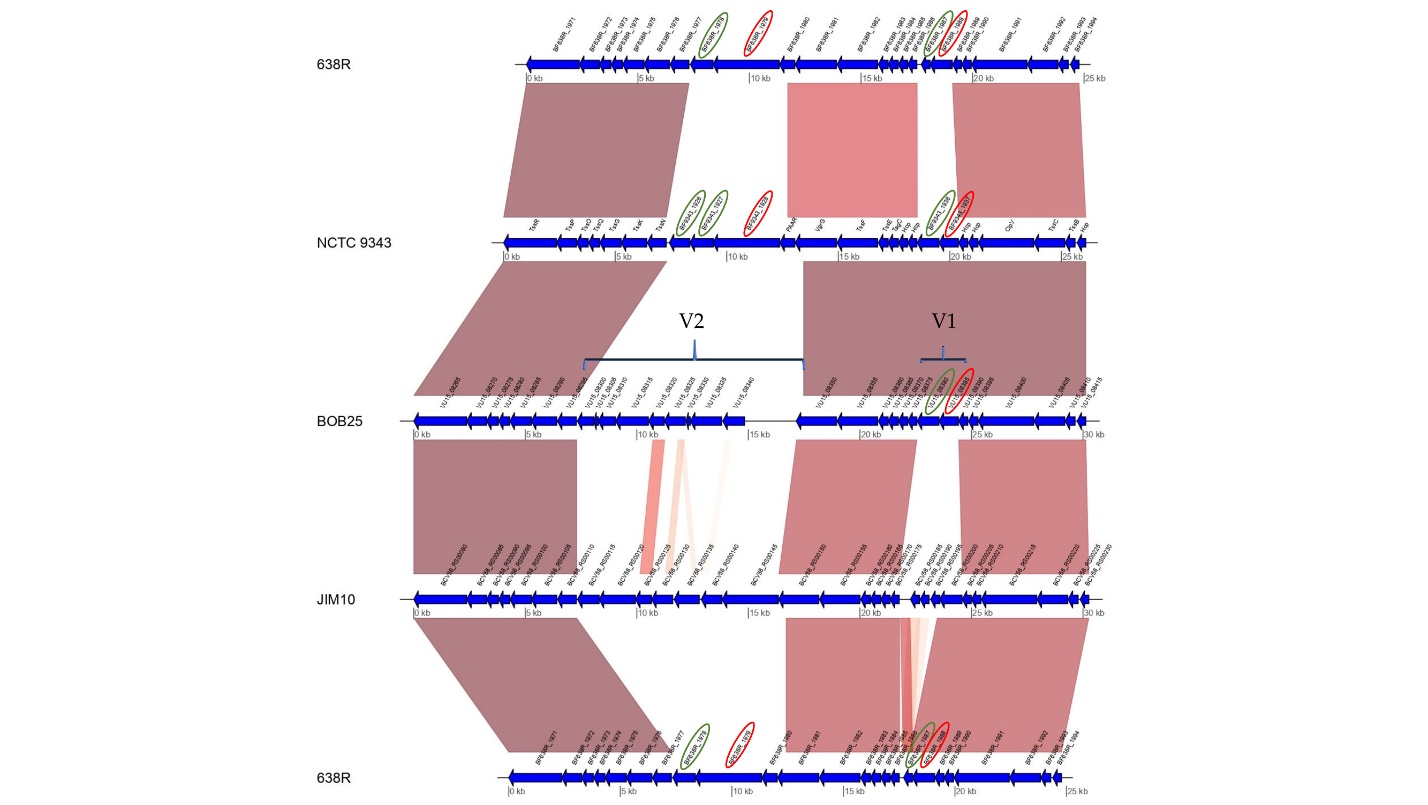


**Figure S1.** Genome comparison of the GA3 locus of the T6SS of the toxigenic strain *B. fragilis* BOB25 with those of the reference strains *B. fragilis* JIM10, *B. fragilis* 638R and *B. fragilis* NCTC 9343. Homologous regions are marked in red; color intensity indicates the degree of coincidence of genomic fragments; variable regions (V1 and V2) are not colored.


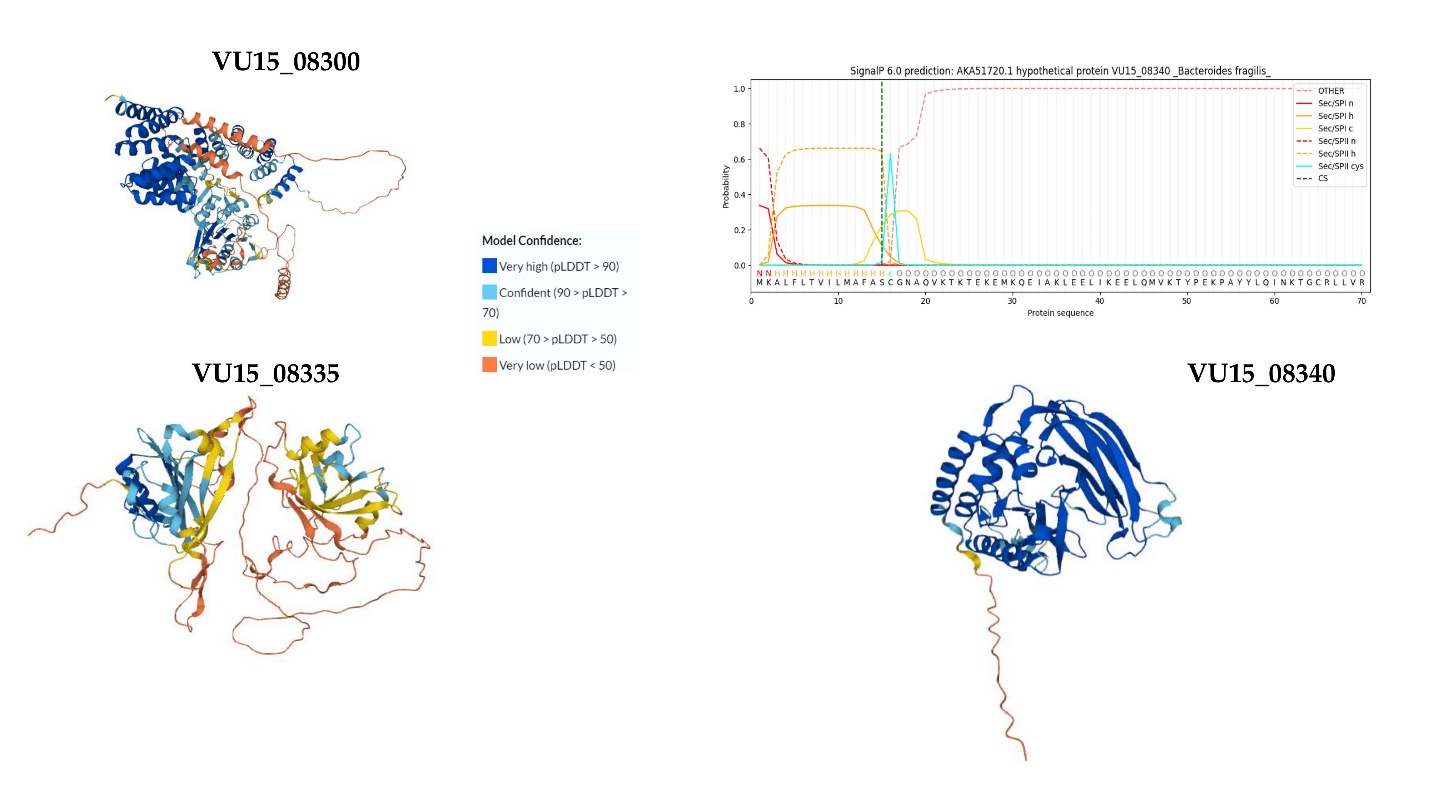


**Figure S2. Figure 3** Functional annotation of the E-I protein pairs in the V2 region. Functional annotation of the AKA51712.1 protein encoded by VU15_08300 revealed CHAT domain-containing protein that appears to be a transmembrane protein. AKA51719.1 protein encoded by VU15_08335 According to the data obtained, the AKA51719.1 protein is completely homologous to lipoprotein A0A2M9V3N9 of *Bf* 12905 and protein R7JKL0, which is closely related to the *Alistipes putredinis* CAG:67 of MGS:67. This protein contains several domains (1-230 and 231-466), one of which is the transmembrane domain. AKA51720.1 protein encoded by the VU15_08340 is a lipoprotein and is highly conserved throughout the *Bacteroides* genus
